# Visual Thinking to Explore “Relational Pharmacology”: Systemic Maps for Managing Non-Selective Antidepressants in Cardiovascular Prevention

**DOI:** 10.3390/pharmacy13040091

**Published:** 2025-06-27

**Authors:** Irene García-Domínguez, Azahara Rodríguez-Luna, Manuel Machuca

**Affiliations:** Departamento Ciencias de la Salud y Biomédicas, Facultad de Ciencias de la Salud, Universidad Loyola Andalucía, 41074 Sevilla, Spain; amrluna@uloyola.es (A.R.-L.); mmachuca@uloyola.es (M.M.)

**Keywords:** relational pharmacology, polypharmacy, systemic maps, medication risks, active learning

## Abstract

Relational pharmacology introduces an innovative approach using visual thinking to understand how drugs interact with multiple body systems, addressing the limitations of the traditional “reductionist approach”. While conventional pharmacology focuses on individual drug effects, it struggles with the complexities of polypharmacy, where multiple medications interact via shared metabolic pathways. This article highlights integrating systemic maps into educational methodologies to empower students in identifying and assessing medication risks. By visualizing the body and drug therapy as interconnected systems, students can better understand complex pharmacological interactions beyond linear frameworks. This approach enables active learning and real-life case analysis, such as cardiovascular prevention with non-selective antidepressants, where multiple drug interactions must be considered. It also fosters global health education by promoting the exchange of effective teaching practices and addressing challenges in healthcare training. Systemic maps prepare students for clinical decision-making by enhancing their ability to manage risks and complex cases effectively.

## 1. Introduction

Advancements in the field of Health Sciences demand that professionals acquire critical thinking, problem-solving, communication, collaboration, and teamwork skills, among other essential competencies [1]. This requires updating curricula to promote critical reasoning, flexibility, adaptability to uncertainty, and self-directed learning. Specifically, pharmacology presents a complex subject for pharmacy students, requiring them to make significant connections between previously studied disciplines such as molecular biology, biochemistry, and physiology to prevent, detect, and solve drug-therapy problems [2,3]. Beyond memorizing isolated facts, students must understand how drugs act on complex biological systems, and how those effects translate into therapeutic outcomes. This detailed study includes pharmacodynamics, which evaluates the effects of medications on various human body systems such as the cardiovascular, nervous, and digestive systems, alongside pharmacokinetics, encompassing how the body absorbs, distributes, metabolizes, and excretes drugs.

Traditionally, teaching pharmacology has focused on analyzing the effects of each drug in isolation, a model that aligns with what can be described as a “reductionist approach” or “single-drug paradigm”. This perspective is especially useful for medicine students, who rely on pharmacology as an important tool to help them in therapeutic decisions, but it does not work when patients take a great amount of medication for different medical conditions, often sharing similar metabolic routes or even mechanisms of action. In real life scenarios, this is often the case, with patients diverging form the straightforward conception of “one drug, one effect”. As a result, it is difficult to manage polypharmacy and the role played by specific receptors to achieve therapeutic goals. This underscores the need for pharmacology teaching to evolve towards approaches that reflect the interconnectedness of physiological systems and the clinical complexity of modern therapy.

Drug-therapy practices such as Medication Management require identifying any deviations from therapeutic goals—whether due to side effects, drug–drug interactions, lack of effectiveness, or inappropriate drug selection. Students therefore need tools that allow them to visualize and interpret what is happening pharmacologically in real patients. The ability to make connections between mechanisms of action and clinical signs or symptoms is essential for safe and effective decision-making. Authors such as Ausubel argue that meaningful learning occurs when new concepts substantively connect to students’ prior knowledge, integrating with relevant aspects of their cognitive structure [4]. This relational approach is aligned with constructivist principles, where knowledge is actively constructed by learners rather than passively received [5]. Students bring pre-existing ideas about various phenomena to the learning process, some of which are ad hoc, and others which are deeply rooted. In summary, deep and meaningful learning happens when students can connect new information with previously acquired knowledge from other subjects, enabling them to establish connections that facilitate the construction of their own understanding [3].

From an instructional standpoint, many medical and pharmacy colleges rely heavily on lectures and slide-based presentations. However, more interactive approaches—such as concept mapping and case-based learning—are gaining ground in health education [6].

Systemic mapping has proven to be a valuable tool in analyzing complex problems across various disciplines, including social sciences, engineering, and management, and realizing the difficulty in understanding the comprehensive medication framework in polypharmacy patients. It serves as a helpful instrument to identify drug-therapy problems such as “pharmacotherapy workup” [7]. Systemic mapping fosters critical thinking by visually organizing interconnected information, which is crucial for healthcare professionals to comprehend pharmacotherapy’s complexity. This approach aids in understanding the underlying connections between side effects, lack of effectiveness, and other drug-therapy problems to facilitate a deeper and more integrated learning process among students and practitioners alike [8]. In this study, we present relational pharmacology as a pedagogical and clinical approach that focuses on the emergent properties and interactions of multiple drugs acting across interconnected physiological systems. It is not a new discipline, but rather a conceptual and methodological shift grounded in clinical pharmacology and constructivist learning theory. In this sense, it is aimed at helping students move beyond fragmented or reductionist drug knowledge towards an integrative understanding of therapy.

This approach is operationalized using systemic maps, which serve as the key instructional tool. While concept mapping is effective for organizing hierarchical knowledge, and pharmacotherapy workups are useful for structured clinical decision-making, systemic maps in the context of relational pharmacology go a step further. They offer a dynamic and integrated visualization of how different drugs interact within a patient’s body—linking mechanisms of action, therapeutic effects, and adverse reactions within a systemic and pathophysiological framework.

What distinguishes this tool from previous methods is its capacity to simultaneously represent multiple pharmacological dimensions (e.g., receptor targets, overlapping adverse effects, redundancies in mechanism, and synergistic effects) in a single visual structure. This allows students to understand not only the individual actions of drugs, but also how their combined effects influence the patient holistically. In doing so, systemic maps enable learners to develop clinical reasoning skills grounded in pharmacodynamic logic, effectively bridging the gap between theoretical knowledge and practical patient care.

Therefore, this article aims to suggest how a teaching tool like systemic maps promotes visual thinking for learning relational pharmacology for pharmacy students, instead of traditional medication assessment models like the reductionist approach represents. We will illustrate this with a complex clinical case of a polypharmacy patient to identify drug-therapy problems that needed to be solved.

## 2. Materials & Methods

### 2.1. Case Description

This paper reassesses a previously documented case of a 74-year-old woman with a history of coronary ischemia [8]. Her physician prescribed her an antidepressant treatment because of the death of one of her sons. After three weeks taking the new medication, she began to report significant drowsiness, tiredness, and dry mouth, specifically in the morning. During self-monitoring of blood pressure (BP), she consistently recorded lower-than-usual values (90–110 mmHg systolic BP and 50–65 mmHg diastolic BP). Due to her history of ischemia and cardiac rhythm disorders, such low BP values were considered suboptimal, especially regarding her other symptoms. Given her cardiac rhythm disorders, an electrocardiogram was conducted and evaluated by a cardiologist via telemedicine, confirming widespread T-wave flattening and ventricular extrasystoles. The patient’s drug therapy is detailed in Table 1, which outlines the medications, dosages, and specific therapeutic actions involved.

### 2.2. Proposed Resolution Methodology

To address the complexity of her medication, two educational tools will be employed. Firstly, traditional pharmacological analysis methods will be utilized, including the compilation of a table detailing the following: medication; pharmacological action; mechanisms of action; interactions; and adverse reactions [9].

Secondly, systemic maps, a teaching tool, will be employed. These maps will include the medication name, key words identifying its mechanism of action, and the main adverse reactions experienced by the patient, all interconnected with arrows and color codes [10]. Medication information will be sourced from the Access Medicine database [11] and complementary sources.

The implementation of the systemic mapping methodology in the classroom followed a progressive and structured instructional sequence, designed to ensure both conceptual understanding and skill development. This course of action involves five phases. The process began with an introductory training phase, during which students were exposed to pre-constructed systemic maps developed by the instructor. These examples were presented and analyzed in class over a period of approximately 2 h, allowing students to become visually and conceptually familiar with the structure, logic, and interpretive strategies involved in systemic mapping.

Following this initial exposure, the instructor conducted a live demonstration on the board, resolving a clinical case step-by-step using the systemic mapping approach (1 h). This session served to model the reasoning process and illustrate how mechanisms of action, therapeutic effects, and adverse reactions could be integrated and visualized in relation to a real patient case.

In the third phase, students actively participated in the construction of systemic maps through a guided group exercise facilitated by the instructor (1 h). This collaborative session allowed students to apply their pharmacological knowledge in context, while receiving formative feedback and clarification in real time.

Subsequently, students engaged in supervised individual practice (2 h), during which they constructed systemic maps based on assigned clinical cases. The instructor provided support and guidance as needed, ensuring that students correctly linked pharmacodynamic actions with clinical manifestations and drug-therapy problems.

Finally, to assess learning outcomes, each student was required to individually construct and submit a complete systemic map as part of a summative evaluation. This exercise included not only the visual diagram, but also a brief written justification of the pharmacological reasoning behind the map. This multistep process ensured that students were trained not only to interpret systemic maps, but also to create them independently, fostering a deeper integration of theoretical and clinical knowledge. To assess the educational impact of this approach, a survey was conducted at the end of the semester. The aim was to explore students’ perspectives on the use of systemic maps—specifically, whether these tools supported their understanding of pharmacology, enhanced their decision-making skills, aided in solving medication-related problems, and helped them feel better prepared for future patient care. The survey was conducted with 45 students from the 3rd year of the Pharmacy degree program.

## 3. Results

### 3.1. Comparison of Learning Methods: Traditional vs. Systemic Mapping

A comparative analysis was conducted to evaluate two approaches to analyzing a clinical case: the traditional method and the systemic mapping approach.

**Figure 1:**
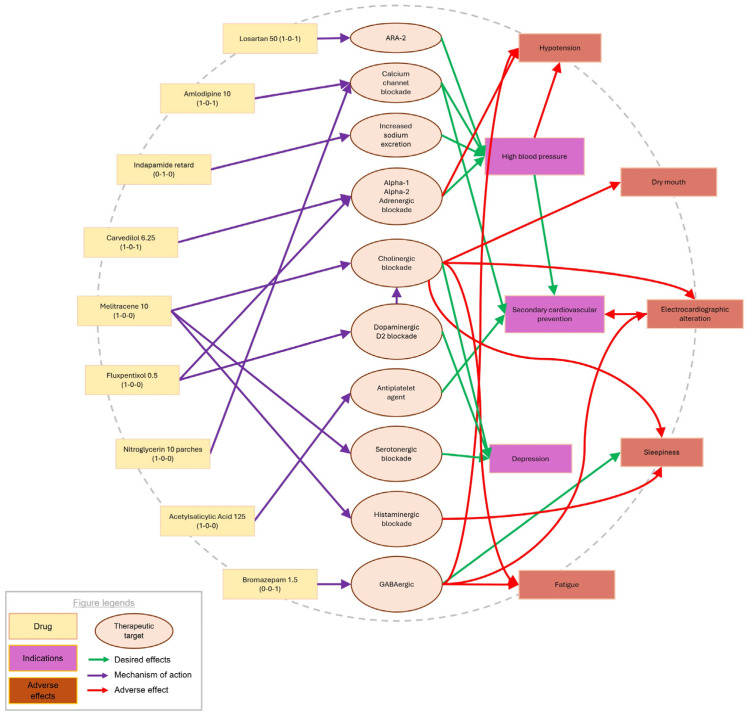
The diagram visually illustrates how each medication is connected to circles containing key terms that describe its mechanisms of action and potential effects. This graphical representation allows for a clear understanding of how the drugs interact with specific biological targets, highlighting both the therapeutic benefits and possible adverse effects. By linking the medication to relevant keywords, it provides an intuitive overview of the pharmacological pathways involved, aiding in a more comprehensive analysis of drug behavior and efficacy.

As previously introduced, systemic maps offer a different analytical framework compared to traditional pharmacological methods. Rather than simply listing mechanisms of action, therapeutic targets, interactions, and adverse effects for each medication—as shown in Table 2—systemic maps (Figure 1) provide an integrated visual representation that connects each drug to its key pharmacodynamic properties, adverse effects, and therapeutic outcomes. This structure supports the development of visual thinking and clinical reasoning, particularly by highlighting overlapping mechanisms and potential interaction points among medications.

### 3.2. Pharmacological Interactions and Clinical Implications

Flupentixol and melitracen are marketed as a fixed-dose combination based on a patent that describes their complementary and synergistic effects on the central nervous system [12].

Following the compilation of Table 2 and an in-depth review of the case, we identified that melitracen is a tricyclic antidepressant that inhibits the reuptake of norepinephrine and serotonin and also has a certain antagonistic effect on histamine and acetylcholine receptors [12,13].

Flupentixol, a neuroleptic that blocks D1 and D2 receptors, along with various serotonin and histamine receptors, and also acts as an alpha-1 adrenergic antagonist [12,13,14,15], could therefore also contribute to the observed antihypertensive effect. This pharmacodynamic overlap may explain the markedly reduced blood pressure values recorded in the patient (90–110 mmHg systolic and 50–65 mmHg diastolic), suggesting a cumulative effect on vascular tone and autonomic regulation, producing an enhanced antihypertensive effect when used concomitantly with melitracen and carvedilol, a coronary vasodilator and antihypertensive indicated for patients with a history of ischemic heart disease. Additionally, the anticholinergic effects of melitracen and flupentixol [12,15] are evident from the patient’s reported dry mouth, especially in the morning, and the drowsiness caused by melitracen’s and flupentixol’s antihistaminic effect, although this is less likely due to the drug’s pharmacokinetics.

Moreover, anticholinergic medications are contraindicated in patients with cardiovascular histories, particularly in the presence of potential arrhythmia. Both tricyclic antidepressants and neuroleptics have been associated with nonspecific electrocardiographic abnormalities, such as ventricular repolarization changes, QT interval prolongation, and widening, flattening, or inversion of the T wave [16,17,18]. These findings were confirmed by electrocardiographic tests, as described in [18].

### 3.3. Insight from Systemic Mapping

Visual assessment through the creation of a systemic map (Figure 1), where each medication is graphically linked with circles containing keywords describing their mechanisms of action and their potential effects, further corroborated these interactions. Graphically designing this map allowed us to observe excessive arrow convergence on a specific action mechanism, such as alpha-adrenergic blockade, and highlighted that both this mechanism and cholinergic blockade are primarily responsible for the patient’s adverse effects. This visual approach enabled us to quickly identify where to focus our investigation.

Upon examining the diagram, we identified that reducing the cholinergic burden on the patient should be a priority. The diagram guided us towards changing the antidepressant to one with a distinct mechanism of action to avoid cumulative adrenergic blockade and prevent excessive hypotension, thereby preserving her baseline antihypertensive treatment. Following this evaluation, the patient was referred to her primary care physician to consider discontinuing melitracen–flupentixol treatment and switching to a selective serotonin reuptake inhibitor without cholinergic or noradrenergic effects, such as citalopram, sertraline, or escitalopram. The physician decided to discontinue melitracen-flupentixol and initiate citalopram instead. The gradual withdrawal of the former medication and the introduction of the new antidepressant alleviated the patient’s somnolence, fatigue, and dry mouth, while stabilizing her blood pressure around 130/75 mmHg.

### 3.4. Student Feedback on the Educational Intervention

**Figure 2:**
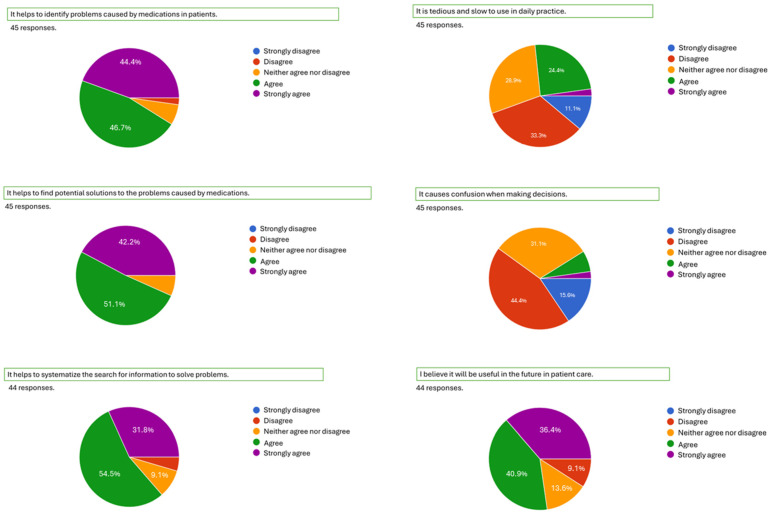
Survey results based on whether the student strongly disagrees, disagrees, neither agrees nor disagrees, agrees, or strongly agrees with the question asked.

As previously described, a survey was conducted at the end of the course to assess the educational impact of the systemic mapping approach. The survey involved 45 third-year pharmacy students who had participated in the complete instructional sequence using systemic maps. The survey results indicate generally positive outcomes. Most students reported that the approach aids in identifying medication-related problems (91.1% agree or strongly agree), supports the development of potential solutions (93.3%), and promotes a systematic method for retrieving information to solve clinical issues (86.3%). They also believe it will be valuable in their future patient care (77.3%). Only a small minority of students (26.7%) found the approach difficult to use or confusing (Figure 2).

## 4. Discussion

### 4.1. Rethinking Pharmacology Education in Complex Clinical Contexts

The pharmacological complexity observed in real-world clinical settings—particularly in patients with multiple comorbidities and polypharmacy—highlights the urgent need to rethink how we educate future pharmacology professionals. Traditional approaches often fall short in preparing students to manage therapeutic decisions involving numerous interacting drugs [1,3]. This reality calls for the incorporation of a relational approach to pharmacology, one that enables students to understand how therapeutic goals are achieved not through isolated drug actions, but through the dynamic interplay of multiple medications within the patient’s physiological context [3,19]. Currently, the importance of reinforcing critical thinking, problem-solving, communication, collaboration, and teamwork skills in students is recognized as a basic concept for teaching discipline from an integrative perspective. Pharmacists today—and in the future—must be equipped to work collaboratively as part of interprofessional healthcare teams. They should serve as patient advocates and demonstrate strong leadership, while delivering care to diverse populations and promoting the health and well-being of both individuals and communities. Additionally, they must be prepared to educate a wide range of audiences and effectively manage complex, technology-driven work environments [20].

Depression is a common disease among the elderly population and in patients who have experienced a cardiovascular event. Strober and Arnett report that its prevalence is 50% in older adults who have suffered a myocardial infarction [21]. In these cases, it is very common to use a great number of medicines to prevent more cardiovascular events and to stabilize depression symptoms. Thus, drug-therapy optimization is a significant challenge due to potential drug–drug interactions between medications used for both conditions, which share neuroreceptors as therapeutic targets and can affect the goals of therapy in terms of effectiveness and safety, which could increase the risk of drug-therapy problems. Too much information needs to be organized [17].

### 4.2. Integration of Visual Learning with Clinical Reasoning

In this case, the use of a systemic map to visualize the mechanism of actions of all the medications used enables the student to understand their real effects in this patient, like an X-ray photograph for a radiologist. This tool not only helps students understand and learn pharmacology in terms of pharmacodynamics but also allows them to understand the best way to solve problems with other healthcare practitioners. A clinical pharmacist today not only ought to know about pharmacology. A new pharmacist must help to solve the complexity we face in the real world of health nowadays [20].

Thus, we propose the concept of “relational pharmacology” and systemic maps as a tool to better understand it, as an approach to pharmacology that shifts from studying each medication used by the patient to understanding that their effects depend not only on the action of each drug but on the resulting combined effect of all the medication used by the patient to achieve the desired goals of therapy.

### 4.3. Implications for Curriculum Design and Healthcare Practice

This approach moves away from the singular view of drug administration as a response to medical diagnoses and is based on a deep understanding of how drugs affect the patient from a molecular, biochemical, and pathophysiological perspective. To achieve this, in many cases, it is essential to add the patient’s subjective experience with medication—including emotional, cognitive, and behavioral dimensions—as described in the concept of “medication experience” [22]. This perspective aligns with new healthcare practices such as Pharmaceutical Care, focused on preventing, detecting, and solving drug-therapy problems with a comprehensive approach [23]. This is an essential perspective for addressing persistent problems such as noncompliance, adverse drug reactions, lack of therapeutic effectiveness, or the need for deprescription. These issues require retrospective evaluation grounded in a biochemical understanding of drug mechanisms of action, their pathophysiological implications, and the patient’s medication experience. In this context, educational strategies gain relevance: greater perceptions of authenticity and alignment in learning tasks, physical contexts, and assessment methods have been associated with deeper learning and the development of transferable, generic skills. When these elements are perceived as more authentic and coherent, students engage more meaningfully with the material, enhancing both their understanding and their ability to apply knowledge in real-world, complex clinical scenarios [24]. Therefore, it is necessary to move beyond traditional teaching methodologies that tend to emphasize the memorization of isolated facts (mechanisms of action, interactions, etc.), thereby limiting meaningful learning for students [3,20,24,25].

### 4.4. Reflections on the Student Learning Experience

According to Ausubel, teaching must be based on constructivist learning theory instead of rote learning, emphasizing the need to connect new knowledge with pre-existing knowledge to facilitate deep and lasting learning [4]. To address these educational challenges, the use of systemic maps as an innovative pedagogical tool is proposed. These maps, actively constructed by students, facilitate the integration of concepts previously learned in areas such as molecular biology or pathophysiology with the principles of pharmacology.

In this way, it seems to be that systemic maps offer significant advantages over traditional learning methods by allowing a substantial connection with students’ prior knowledge, associating concepts related to previously studied subjects. Additionally, visualizing the complexity of the case facilitates the memorization and understanding of the information presented.

For all these reasons, this methodology not only improves learning due to its visual nature and the active participation of students in the process, but also promotes deep and integrated understanding. This visual technique facilitates the ability to establish meaningful connections between different concepts, thereby enhancing deep and more effective learning.

### 4.5. Limitations and Challenges of Implementing Systemic Mapping

While systemic maps offer clear pedagogical and clinical benefits, their implementation is not without challenges. It is important to recognize the limitations of this methodology to ensure its effective integration into pharmacology curricula and to guide future research and improvements. A critical evaluation of these limitations also reinforces the academic rigor and transparency of the approach proposed in this study.

Firstly, the construction of systemic maps requires significant time and effort from both students and educators. This process involves a deep cognitive engagement that may be perceived as complex or overwhelming, especially during the early stages of training, when students are still building their foundational knowledge in pharmacology, pathophysiology, and molecular biology.

Secondly, this methodology demands specific training and preparation from instructors to effectively guide the construction and analysis of these maps. Not all educators are familiar with constructivist approaches or with visual tools that integrate multiple disciplines, which can pose challenges in more traditional academic environments.

Furthermore, although systemic maps are effective in illustrating pharmacodynamic interactions, they may oversimplify certain clinical nuances or fail to capture the full complexity of patient-specific variables. The inherently schematic nature of these tools can obscure the complexity of patient-specific variables, such as comorbidities, pharmacogenetic differences, or psychosocial factors, unless carefully contextualized within a broader clinical discussion.

## 5. Conclusions

In summary, systemic maps are a tool that offer students a relational perspective, allowing them to identify, in a structured manner, the reasons and potential impacts of drug interactions or adverse effects experienced by the patient. They also facilitate the detection and resolution of drug-therapy problems. This comprehensive approach aims to provide a deeper and more holistic understanding of the patient’s current pharmacological therapy, thereby enabling more informed and personalized therapeutic decisions. This has been upheld by the survey results, which highlight the effectiveness of systemic maps in enhancing students’ understanding and application of pharmacological concepts in clinical scenarios.

## Figures and Tables

**Table 1:** Comprehensive overview of the patient’s pharmacological treatment regimen, listing each medication alongside its strength, dosage, and timing relative to meals, specifically at breakfast, lunch, and dinner. Additionally, the table specifies the indication or pathology for which each drug is prescribed, and the onset of the treatment, giving a clear and structured presentation of the care plan.

Medication	Strength	Dosage	Breakfast	Lunch	Dinner	Indication/Pathology	Treatment Onset
Losartan	50 mg	1 tablet twice a day	1	0	1	Hypertension (Angiotensin II receptor blocker)	Initiated 9 months ago
Amlodipine	10 mg	1 tablet once a day	0	0	1	Hypertension (calcium channel blocker)	Initiated 9 months ago
Indapamide retard	1.5 mg	1 tablet once a day	0	1	0	Hypertension (diuretic)	Initiated 9 months ago
Carvedilol	6.25 mg	1 tablet twice a day	1	0	1	Cardiovascular risk prevention (alpha-1 and beta-blocker)	Initiated 9 months ago
Acetylsalicylic Acid	125 mg	1 tablet once a day	1	0	0	Cardiovascular risk prevention (NSAID, antiplatelet agent)	Initiated 2 years ago
Nitroglycerine	10 mg	1 patch a day	1	0	0	Cardiovascular risk prevention (nitrate, vasodilator)	Initiated 9 months ago
Melitracen-flupentixol	10/0.5 mg	1 tablet once a day	1	0	0	Tricyclic antidepressant + neuroleptic agent	Initiated 3 weeks ago
Bromazepam	1.5 mg	1 tablet once a day	0	0	1	Benzodiazepine por anxiety and insomnia	Initiated 1.5 months ago

**Table 2:** The patient’s current drug-therapy is constructed using traditional methodology. This table includes information retrieved from the Access Medicine database, detailing the medication name, pharmacological action, mechanism of action, pharmacological interactions, and contraindications.

Medication	Pharmacological Action	Mechanism of Action	Interactions	Contraindications
Losartan	HTN	Angiotensin II receptor blockers	May cause hypercalcemia in patients receiving potassium-sparing diuretics, aldosterone antagonists, or angiotensin receptor blockers, or heparin, trimethoprim, potassium supplements, and potassium salt substitutes. Starting dose should be reduced in elderly patients. Risks of hypotension using tricyclic antidepressants. Kidney function must be monitored	Renal insufficiency (GFR < 60 mL/min/1.73 m^2^).
Amlodipine	Hypertension	Calcium channel blockers	Risks of hypotension, edema in pretibial area	Enhances the effect of other antihypertensives such as beta-blockers, ACE, alpha-adrenergic antagonists and diuretics.
Indapamide retard	Diuretic	Diminish sodium reabsorption at different sites at the nephron, increasing sodium and water losses	Risk of arrhythmias and torsade de pointes with some tricyclic’s antidepressants, antipsychotics, and beta-blockers K. Risk of hypotension with tricyclic’s antidepressants.	Renal insufficiency, risks of hypokalemia
Carvedilol	Antihypertensive and coronary vasodilator	Alpha-1 and beta-blocker	Increase plasma levels of SSRI. Risk of bradycardia with some MAO inhibitors.	Atrioventricular block, cardiogenic shock, Chronic Obstructive Pulmonary Disease, Asthma.
Acetylsalicylic acid	Antiplatelet agent	COX1 and COX2 inhibitor	Caution with SSRI use due to hemorrhage risks. Can also diminish ARA-II and beta-blockers’ effectiveness	Gastrointestinal bleeding
Nitroglycerine	Vascular smooth muscle dilator	Smooth muscle relaxant. Calcium levels in the vascular myocyte decrease due to inhibition of calcium ion entry or promotion of its exit, ultimately resulting in vasodilation.	Hypotension risks with calcium channel blockers, beta-blockers, ACE, tricyclic antidepressants, and neuroleptic agents	Anemia, myocardial insufficiency
Melitracen-flupentixol	Melitracen: tricyclic antidepressant. Flupentixol: antipsychotic.	Melitracen: inhibitor of norepinephrine, and serotonin reuptake, histamine, and acetylcholine blocker. Flupentixol: Antagonism at alpha-1 adrenergic, D1 and D2 dopaminergic, serotonergic, histaminergic, and muscarinic acetylcholine receptors.	Toxicity risk increases with central nervous system depressants like anticholinergics. Melitracen increases cardiovascular effects of adrenaline and noradrenaline	Recent myocardial infarction, atrioventricular block. Intoxication risks with central nervous system depressants
Bromazepam	Anxiolytic	GABA activity increase, by facilitating its binding to the GABAergic receptor.	Toxicity risks with anxiolytics, antipsychotics, antidepressants, antihistamines, etc.	Apnea, respiratory depression, myasthenia gravis

Note: HTN Hypertension, ACE Angiotensin-Converting Enzyme, GFR Glomerular Filtration Rate, ARA-II Angiotensin II Receptor Antagonists, SSRIs selective serotonin reuptake inhibitors, GABA Gamma-Aminobutyric Acid, MAO Monoamine Oxidase, COX Cyclooxygenase.

## Data Availability

The original contributions presented in this study are included in the article. Further inquiries can be directed to the corresponding author(s).
